# Impact of host- and early treatment-related factors on mortality in ICU patients with candidemia: a bicentric retrospective observational study

**DOI:** 10.1186/s40560-020-00450-7

**Published:** 2020-04-26

**Authors:** Shingo Ohki, Nobuaki Shime, Tadashi Kosaka, Naohisa Fujita

**Affiliations:** 1 Department of Emergency and Critical Care Medicine, Graduate School of Biomedical and Health Sciences, Hiroshima University, 1-2-3 Kasumi, Minami-ku, Hiroshima, 734-8551 Japan; 2 Department of Pharmacy, Kyoto Prefectural University of Medicine, Kajii-cho, Kawaramachi-Hirokoji, Kamigyo-ku, Kyoto, 602-8566 Japan; 3 Department of Infection Control and Laboratory Medicine, Kyoto Prefectural University of Medicine, Kajii-cho, Kawaramachi-Hirokoji, Kamigyo-ku, Kyoto, 602-8566 Japan

**Keywords:** *Candida*, Candidemia, Intensive care unit, Prognostic factor, Antifungal therapy, CVC removal, Mortality

## Abstract

**Background:**

Candidemia is one of the most life-threatening infections among critically ill patients in the intensive care unit. However, the number of studies on the impact of host- and early treatment-related factors on mortality in this cohort is limited. The aim of this study was to investigate the relationship between clinically relevant factors, including early treatment (appropriate antifungal therapy and/or central venous catheter removal) and mortality in intensive care unit patients with candidemia.

**Methods:**

We performed a retrospective observational study in two Japanese University hospitals between January 2007 and December 2016. Adult intensive care unit patients with candidemia who met the following inclusion criteria: (1) ≥ 18 years old; (2) admitted in intensive care unit at the time of onset; and (3) central venous catheter in situ at the time of onset were included. We performed univariate and multivariate logistic regression analysis to identify factors associated with 30-day crude mortality.

**Results:**

A total of 68 patients met the inclusion criteria, 47 (69%) of whom were males. The median age was 68.0 (interquartile range, 61.0–76.0) years. The most common causative *Candida* species was *Candida albicans* (40 [59%] patients). With respect to the source of infection, central venous catheter-related candidemia was the most frequent (30 [44%] patients). Thirty-day crude mortality was 54% (37 patients). In multivariate logistic regression analysis, Acute Physiology and Chronic Health Evaluation II score (1-point increments) was the only factor that was independently associated with higher 30-day crude mortality. Other variables, including appropriate antifungal therapy and/or central venous catheter removal ≤ 24 h and ≤ 48 h following onset, did not significantly influence mortality.

**Conclusions:**

Candidemia in intensive care unit patients is still associated with high 30-day crude mortality rates. The only predictor of death was Acute Physiology and Chronic Health Evaluation II score ≤ 24 h following candidemia onset. Early empiric antifungal therapy and/or early CVC removal conferred no significant clinical benefit on survival in this patient population.

## Introduction

*Candida* species are among the most common causative agents of nosocomial bloodstream infections [1]. In critically ill intensive care unit (ICU) patients, the incidence rate of *Candida* bloodstream infection (candidemia) is estimated at between 2.1 and 6.9 cases per 1000 ICU admissions [2–4], with a high crude mortality rate of 43–61% [2–5].

To date, many studies have investigated factors associated with mortality among candidemia patients across several clinical settings. However, most of the studies included non-ICU patients and therefore, little is known about prognostic factors in ICU patients with candidemia. Specifically, a limited number of studies have investigated the impact of early appropriate antifungal therapy and/or early removal of central venous catheter (CVC) on mortality, with inconsistent results [2–6].

Consequently, in this study, we investigated the relationship between clinically relevant factors, including early treatment (appropriate antifungal therapy and/or CVC removal) and mortality in ICU patients with candidemia.

## Methods

### Study design and patient selection

We conducted a bicentric, retrospective observational study in two university hospitals in Japan between January 2007 and December 2016. Participating hospitals included the Hiroshima University Hospital (746 beds) and the University Hospital, Kyoto Prefectural University of Medicine (1065 beds). We used the microbiological database of each participating hospital to identify positive blood cultures for *Candida* species. We included adult ICU patients with candidemia who met the following inclusion criteria: (1) were ≥ 18 years old; (2) were admitted in the ICU at the time of onset; and (3) had a CVC in situ at the time of onset.

All data were anonymized, and the requirement for informed consent was waived due to the retrospective study design. This study was approved by the institutional review board of Hiroshima University (approval number: E-746) and Kyoto Prefectural University of Medicine (approval number: ERB-C-1162).

### Patient variables and outcomes

The following information was collected from each patient’s medical record for covariates: age at onset; sex; time from hospital admission to onset; Acute Physiology and Chronic Health Evaluation (APACHE) II score ≤ 24 h following onset; comorbidities and prior treatment exposure (solid organ malignancy ≤ 1 year before onset, hematological malignancy ≤ 1 year before onset, previous solid organ transplantation, previous hematopoietic stem cell transplantation, liver disease, diabetes mellitus, autoimmune disease, neutropenia, abdominal surgery ≤ 3 months before onset, invasive mechanical ventilation at the time of onset, renal replacement therapy ≤ 30 days before onset, total parenteral nutrition [TPN] at the time of onset, antibiotic therapy [for ≥ 3 days] ≤ 30 days before onset, antifungal therapy [for ≥ 3 days] ≤ 30 days before onset, immunosuppressive therapy ≤ 30 days before onset); shock ≤ 24 h following onset; concurrent bacteremia at onset; breakthrough candidemia; causative *Candida* species; source of candidemia; treatment ≤ 24 h following onset (appropriate antifungal therapy alone, CVC removal alone, or a combination of them [combined intervention]); and treatment ≤ 48 h following onset.

The primary outcome of this study was 30-day crude mortality.

### Definitions

Candidemia was defined as the isolation of *Candida* species from at least one blood culture. Candidemia onset was defined as the time when the first positive blood culture for *Candida* species was drawn from the patient. Neutropenia was defined as an absolute neutrophil count < 500 cells/mm^3^. Immunosuppressive therapy was defined as the administration of corticosteroids, chemotherapy drugs, or other immunosuppressive drugs. Shock was defined as the initiation or increment of inotropes/vasopressors ≤ 24 h following onset to maintain a mean arterial blood pressure of ≥ 65 mmHg. Breakthrough candidemia was defined as candidemia that occurred in patients receiving antifungal agents at the time of onset for ≥ 3 days. CVC-related candidemia was defined as the isolation of the same *Candida* species from blood culture and CVC tip culture. Appropriate antifungal therapy was defined as the administration of the correct dose of antifungal agent for a susceptible *Candida* species. Correct doses of antifungal agents were defined as follows: caspofungin, loading dose of 70 mg, then 50 mg daily; micafungin, 100–150 mg daily; liposomal amphotericin B, 2.5–5 mg/kg daily; fluconazole, loading dose of 800 mg (or 12 mg/kg), then 400 mg (or 6 mg/kg) daily; fosfluconazole, loading dose of 800 mg (or 12 mg/kg) daily for 2 days, then 400 mg (or 6 mg/kg) daily; and voriconazole, loading dose of 400 mg (or 6 mg/kg) twice daily for 2 doses, then 200–300 mg (or 3–4 mg/kg) twice daily [7, 8]. Antifungal susceptibility was determined using the guidelines in the Clinical and Laboratory Standard Institute document M27-S3 [9].

Candidemia occurring > 30 days following the preceding candidemia onset was considered as a new episode. We only included the first episode of candidemia during the study period in the analyses.

### Microbiological procedures

In both participating hospitals, *Candida* species were isolated using an automated blood culture system (BACT/ALERT 3D; bioMérieux, Tokyo, Japan) and identified using the VITEK 2 YST ID card (bioMérieux).

### Statistical analysis

Qualitative variables were reported as frequencies and percentages. Quantitative variables were reported as means and standard deviations (SDs) when parametric, or as medians and interquartile ranges (IQRs) when nonparametric. Qualitative variables were analyzed using Fisher’s exact test. Parametric data were analyzed using the *t* test, and nonparametric data were analyzed using the Mann-Whitney *U* test. We performed univariate and multivariate logistic regression analysis to identify factors associated with 30-day crude mortality. We entered variables significant by univariate logistic regression analysis at the *P* < 0.10 level, as well as prespecified variables, into the multivariate logistic regression analysis. The following prespecified variables were selected on the basis of the results of previous studies and our clinical interest: APACHE II score ≤ 24 h following onset; treatment ≤ 24 h and ≤ 48 h following onset (appropriate antifungal therapy alone, CVC removal alone, or combined intervention). To assess the impact of these two different periods between the time of onset and intervention, we structured two multivariate logistic regression models. Furthermore, we conducted subgroup analyses for factors associated with mortality in patients with CVC-related and CVC-unrelated candidemia. All statistical tests were two-tailed and *P* values < 0.05 were considered statistically significant.

## Results

### Patient characteristics at candidemia onset

We identified a total of 68 patients with a median age of 68.0 years (61.0–76.0) who met the inclusion criteria (Table 1). Of these, 47 (69%) were males. The most common comorbidities were liver disease, diabetes mellitus, and autoimmune disease (for each, 14 [21%] patients). Fifty-six (82%) patients were receiving invasive mechanical ventilation at the time of onset. Within 30 days prior to onset, 65 (96%) patients had received antibiotics for ≥ 3 days.

**Table 1:** Patient characteristics at candidemia onset

Variables	Total cohort (*n* = 68)	30-day survivors (*n* = 31)	30-day non-survivors (*n* = 37)	*P* value
Male sex	47 (69.1)	22 (71.0)	25 (67.6)	0.798
Age (years)	68.0 (61.0–76.0)	68.0 (58.0–76.0)	68.0 (63.0–76.0)	0.782
Time from hospital admission to onset (days)	24.5 (8.0–46.5)	20.0 (6.0–42.0)	26.0 (9.0–47.0)	0.479
APACHE II score (points)	21.0 (18.0–26.0)	19.0 (13.0–24.0)	23.0 (19.0–27.0)	0.003
Comorbidities and prior treatment exposure				
Solid organ malignancy*	11 (16.2)	6 (19.4)	5 (13.5)	0.531
Hematological malignancy*	2 (2.9)	0 (0.0)	2 (5.4)	0.496
Solid organ transplantation	4 (5.9)	1 (3.2)	3 (8.1)	0.620
Hematopoietic stem cell transplantation	1 (1.5)	0 (0.0)	1 (2.7)	1.000
Liver disease	14 (20.6)	6 (19.4)	8 (21.6)	1.000
Diabetes mellitus	14 (20.6)	4 (12.9)	10 (27.0)	0.229
Autoimmune disease	14 (20.6)	7 (22.6)	7 (18.9)	0.769
Neutropenia (< 500 cells/mm^3^)	2 (2.9)	0 (0.0)	2 (5.4)	0.496
Abdominal surgery**	18 (26.5)	7 (22.6)	11 (29.7)	0.587
Invasive mechanical ventilation	56 (82.4)	23 (74.2)	33 (89.2)	0.124
Renal replacement therapy^†^	23 (33.8)	7 (22.6)	16 (43.2)	0.122
Total parenteral nutrition	43 (63.2)	18 (58.1)	25 (67.6)	0.458
Antibiotic therapy (for ≥ 3 days) ^†^	65 (95.6)	30 (96.8)	35 (94.6)	1.000
Antifungal therapy (for ≥ 3 days) ^†^	6 (8.8)	2 (6.5)	4 (10.8)	0.681
Immunosuppressive therapy^†^	26 (38.2)	9 (29.0)	17 (46.0)	0.211
Shock	42 (61.8)	17 (54.8)	25 (67.6)	0.324
Concurrent bacteremia	22 (32.4)	10 (32.3)	12 (32.4)	1.000
Breakthrough candidemia	6 (8.8)	2 (6.5)	4 (10.8)	0.681

Values are given as *n* (%) or median (interquartile range). *APACHE* acute physiology and chronic health evaluation.

*≤ 1 year before onset

**≤ 3 months before onset

^†^≤ 30 days before onset

### Causative Candida species and source of infection

The most common causative *Candida* species was *Candida albicans* (40 [59%] patients), followed by *Candida glabrata* (17 [25%] patients) (Table 2). Two causative *Candida* species were isolated simultaneously in six (9%) patients. With regard to the source of infection, CVC-related candidemia was the most frequent (30 [44%] patients), followed by intra-abdominal infection (10 [15%] patients).

**Table 2:** Causative *Candida* species and source of infection

Variables	Total cohort (*n* = 68)	30-day survivors (*n* = 31)	30-day non-survivors (*n* = 37)	*P* value
Causative *Candida* species*				
*Candida albicans*	40 (58.8)	19 (61.3)	21 (56.8)	0.806
*Candida glabrata*	17 (25.0)	6 (19.4)	11 (29.7)	0.405
*Candida parapsilosis*	8 (11.8)	3 (9.7)	5 (13.5)	0.719
*Candida tropicalis*	7 (10.3)	3 (9.7)	4 (10.8)	1.000
*Candida krusei*	1 (1.5)	1 (3.2)	0 (0.0)	0.456
Others	1 (1.5)	0 (0.0)	1 (2.7)	1.000
Source of infection				
CVC-related	30 (44.1)	15 (48.4)	15 (40.5)	0.625
Intra-abdominal	10 (14.7)	4 (12.9)	6 (16.2)	0.745
Skin and soft tissue	4 (5.9)	3 (9.7)	1 (2.7)	0.324
Cardiovascular	1 (1.5)	0 (0.0)	1 (2.7)	1.000
Others or unknown	23 (33.8)	9 (29.0)	14 (27.8)	0.607

Values are given as *n* (%). *CVC* central venous catheter

*Two causative *Candida* species were isolated simultaneously in 6 (8.8%) patients

### Management of candidemia

For initial antifungal treatment (including inappropriate antifungal treatment), micafungin was most frequently used antifungal agent (in 41 [60%] patients), followed by liposomal amphotericin B (in 11 [16%] patients) (Table 3). Within 48 h following onset, 9 (13%) patients received appropriate antifungal therapy alone, 18 (27%) patients had CVC removal alone, 16 (24%) patients received combined intervention, whereas 25 (37%) patients received either no treatment or inappropriate antifungal therapy alone. In addition, no patient received source control other than CVC removal ≤ 48 h following onset.

**Table 3:** Management of candidemia

Variables	Total cohort (*n* = 68)	30-day survivors (*n* = 31)	30-day non-survivors (*n* = 37)	*P* value
Initial antifungal agent*				
Micafungin	41 (60.3)	19 (61.3)	22 (59.5)	1.000
Liposomal amphotericin B	11 (16.2)	6 (19.4)	5 (13.5)	0.531
Fluconazole	7 (10.3)	2 (6.5)	5 (13.5)	0.442
Voriconazole	3 (4.4)	2 (6.5)	1 (2.7)	0.588
None during candidemia	7 (10.3)	2 (6.5)	5 (13.5)	0.442
Treatment ≤ 24 h following onset				
Appropriate antifungal therapy alone	4 (5.9)	3 (9.7)	1 (2.7)	0.324
CVC removal alone	19 (27.9)	9 (29.0)	10 (27.0)	1.000
Combined intervention	5 (7.4)	2 (6.5)	3 (8.1)	1.000
Treatment ≤ 48 h following onset				
Appropriate antifungal therapy alone	9 (13.2)	3 (9.7)	6 (16.2)	0.494
CVC removal alone	18 (26.5)	8 (25.8)	10 (27.0)	1.000
Combined intervention	16 (23.5)	9 (29.0)	7 (18.9)	0.396

Values are given as *n* (%). *CVC* central venous catheter

*Including antifungal agents used in inappropriate antifungal therapy

### Outcome and factors associated with mortality

Thirty-day crude mortality was 54% (37 patients). In univariate analysis for mortality, the following two variables showed *P* values < 0.10: APACHE II score ≤ 24 h following onset (1-point increments) (odds ratio [OR] 1.14, 95% confidence interval [CI] 1.04–1.25, *P* = 0.005); and renal replacement therapy ≤ 30 days before onset (OR 2.61, 95% CI 0.90–7.57, *P* = 0.077). In multivariate logistic regression analysis, only APACHE II score (1-point increments) was independently associated with higher 30-day crude mortality both in model 1 (OR 1.14, 95% CI 1.03–1.25, *P* = 0.007) and in model 2 (OR 1.14, 95% CI 1.04–1.26, *P*= 0.008) (Table 4). Other variables, including treatment ≤ 24 h and ≤ 48 h following onset, did not significantly influence mortality.

**Table 4:** Multivariate logistic regression analysis of risk factors for 30-day mortality

Variables	Model 1		Model 2	
	OR (95% CI)	*P* value	OR (95% CI)	*P* value
APACHE II score (1-point increments)	1.14 (1.03–1.25)	0.007	1.14 (1.04–1.26)	0.008
Renal replacement therapy*	2.96 (0.91–9.61)	0.070	2.77 (0.87–8.80)	0.085
Treatment ≤ 24 h following onset				
None or inappropriate antifungal therapy alone	Reference			
Appropriate antifungal therapy alone	0.23 (0.02–3.12)	0.267		
CVC removal alone	0.97 (0.29–3.29)	0.961		
Combined intervention	1.66 (0.17–16.56)	0.666		
Treatment ≤ 48 h following onset				
None or inappropriate antifungal therapy alone			Reference	
Appropriate antifungal therapy alone			1.06 (0.17–6.50)	0.948
Source control alone			1.15 (0.30–4.43)	0.840
Combined therapy			0.59 (0.14–2.39)	0.456

*APACHE* acute physiology and chronic health evaluation, *CI* confidence interval, *OR* odds ratio

*≤ 30 days before onset

### Subgroup analysis in patients with CVC-related/-unrelated candidemia

Mortality rates did not differ significantly between patients with CVC-related candidemia (30 patients) and CVC-unrelated candidemia (38 patients) (50% vs 58%, *P* = 0.625). Univariate logistic regression analysis for mortality showed that the following host-related factors were significantly associated with poor 30-day survival: renal replacement therapy ≤ 30 days before onset (OR 12.25, 95% CI 1.27–118.36, *P* = 0.030) in CVC-related candidemia; APACHE II score ≤ 24 h following onset (1-point increments) (OR 1.17, 95% CI 1.03–1.33, *P* = 0.017); and TPN at onset (OR 4.37, 95% CI 1.07–17.79, *P* = 0.039) in CVC-unrelated candidemia. There was no significant association between early intervention (i.e., appropriate antifungal therapy and/or CVC removal) and mortality in the two subgroups. Due to the small sample size, we could not conduct multivariate logistic regression analysis among the subgroups.

## Discussion

In this bicentric observational study, we investigated the characteristics and impact of host- and early treatment-related factors in ICU patients with candidemia. We found that APACHE II score ≤ 24 h following candidemia onset was the only parameter independently associated with death, and that none of the therapeutic interventions (including early empiric antifungal therapy and/or early CVC removal) had a significant impact on mortality. In subgroup analysis, we also found that the other two host-related factors (i.e., renal replacement therapy ≤ 30 days before onset in patients with CVC-related candidemia and TPN at onset in CVC-unrelated candidemia) but not early treatment-related factors were significantly associated with mortality in univariate logistic regression analyses, which supported the primary results.

According to the current published guidelines, prompt antifungal therapy and CVC removal are encouraged [8, 10]. There have been several positive results regarding the effect of early intervention on mortality in ICU patients with candidemia [4, 5, 11]. One study in Spanish ICUs reported that combined intervention (i.e., appropriate antifungal therapy and CVC removal) ≤ 48 h following onset was significantly associated with lower early (≤ 7 days) crude mortality, but not with late (8–30 days) crude mortality [5]. Another study in Indian ICUs demonstrated a significant association between early intervention and 30-day crude mortality [4]. Surprisingly, the results from the present study are not in line with these findings. Differences in patients’ age, background, proportion of CVC placement, or severity of illness among other studies and the present one might account for these discrepancies.

Conversely, and in line with findings from the present study, several studies reported no significant relationships between early intervention and mortality in ICU patients with candidemia [2, 12, 13]. These studies consistently suggested that host-related factors (e.g. comorbidities, prior treatment exposure, or APACHE II score at diagnosis) had more impact on mortality than early intervention. Compared with other patients with bacteremia, patients with candidemia are often more severely ill, receive more organ support therapies (e.g., mechanical ventilation or vasopressors), and are associated with higher mortality rates [14–16]. Therefore, findings from the present and previous studies might be indicative that early interventions in ICU patients with candidemia have less impact on mortality compared to other ICU patients with bacteremia.

We should be aware that all aforementioned studies, including the present one, were observational in design, possibly containing various biases and unmeasured potential confounders. No meta-analysis or randomized trials have yet been conducted to investigate the effect of early intervention in ICU patients with candidemia [17]. In the future, randomized studies targeting subgroups such as patients with septic shock due to candidemia could be considered, as several observational studies with positive results were derived from this population in the past [18, 19].

Our study has a strength in targeting patients with (1) microbiologically confirmed candidemia, (2) onset of candidemia during ICU stay, and (3) CVC in situ at the time of onset, in order to appropriately evaluate the effect of therapeutic intervention. On the other hand, this study had several limitations. Firstly, we could not assess candidemia-attributable mortality, which has been previously reported as 5–22% [4, 11, 20]. However, there has been no accepted definition of attributable mortality and several studies even concluded that candidemia itself did not significantly increase mortality [11, 13]. Secondly, we could not perform subgroup analysis based on causative *Candida* species. According to a previous study, distribution of isolated *Candida* species among invasive candidiasis depends on various factors such as geography, underlying disease, and prior exposure to antibiotics [21]. Further large-scale studies to assess whether clinical features and effects of therapeutic interventions depend on causative *Candida* species are warranted [22]. Acknowledging these strengths and limitations, our study could demonstrate no significant clinical benefit of early intervention among ICU patients with candidemia and suggested the need to conduct a meta-analysis or randomized trial that would potentially impact future clinical practice guidelines.

## Conclusions

Candidemia in ICU patients is still associated with high 30-day crude mortality rates. The only predictor of death was APACHE II score ≤ 24 h following candidemia onset. Early empiric antifungal therapy and/or early CVC removal conferred no significant clinical benefit on survival in this patient population.

## Abbreviations

ICU: Intensive care unit
CVC: Central venous catheter
APACHE: Acute Physiology and Chronic Health Evaluation
TPN: Total parenteral nutrition
SD: Standard deviation
IQR: Interquartile range
